# Antifibrotic role of vascular endothelial growth factor in pulmonary fibrosis

**DOI:** 10.1172/jci.insight.92192

**Published:** 2017-08-17

**Authors:** Lynne A. Murray, David M. Habiel, Miriam Hohmann, Ana Camelo, Huilan Shang, Yang Zhou, Ana Lucia Coelho, Xueyan Peng, Mridu Gulati, Bruno Crestani, Matthew A. Sleeman, Tomas Mustelin, Meagan W. Moore, Changwan Ryu, Awo D. Osafo-Addo, Jack A. Elias, Chun G. Lee, Buqu Hu, Jose D. Herazo-Maya, Darryl A. Knight, Cory M. Hogaboam, Erica L. Herzog

**Affiliations:** 1 MedImmune Ltd., Cambridge, England, United Kingdom.; 2 Department of Medicine, Cedars-Sinai Medical Center, Los Angeles, California, USA.; 3 Department of Pathology, University of Michigan Medical School, Ann Arbor, Michigan, USA.; 4 Yale University School of Medicine, New Haven, Connecticut, USA.; 5 APHP, Hôpital Bichat, Service de Pneumologie A, Centre de Compétences des Maladies Pulmonaires Rares, Paris, France Université Paris Diderot, Sorbonne Paris Cité, INSERM Unité 1152, Paris.; 6 MedImmune LLC., Gaithersburg, Maryland, USA.; 7 Warren Alpert School of Medicine, Providence, Rhode Island, USA.; 8 Viva program, Hunter Medical Research Institute, Newcastle, NSW, Australia.; 9 Department of Anesthesiology, Pharmacology and Therapeutics, University of British Columbia, Vancouver, British Columbia, Canada.; 10 School of Biomedical Sciences and Pharmacy, The University of Newcastle, Callaghan, NSW, Australia.

**Keywords:** Inflammation, Pulmonology

## Abstract

The chronic progressive decline in lung function observed in idiopathic pulmonary fibrosis (IPF) appears to result from persistent nonresolving injury to the epithelium, impaired restitution of the epithelial barrier in the lung, and enhanced fibroblast activation. Thus, understanding these key mechanisms and pathways modulating both is essential to greater understanding of IPF pathogenesis. We examined the association of VEGF with the IPF disease state and preclinical models in vivo and in vitro. Tissue and circulating levels of VEGF were significantly reduced in patients with IPF, particularly in those with a rapidly progressive phenotype, compared with healthy controls. Lung-specific overexpression of *VEGF* significantly protected mice following intratracheal bleomycin challenge, with a decrease in fibrosis and bleomycin-induced cell death observed in the VEGF transgenic mice. In vitro, apoptotic endothelial cell–derived mediators enhanced epithelial cell injury and reduced epithelial wound closure. This process was rescued by VEGF pretreatment of the endothelial cells via a mechanism involving thrombospondin-1 (TSP1). Taken together, these data indicate beneficial roles for VEGF during lung fibrosis via modulating epithelial homeostasis through a previously unrecognized mechanism involving the endothelium.

## Abstract 2

Elevated VEGF is associated with less severe disease in IPF patients, and VEGF overexpression ameliorates bleomycin-induced lung fibrosis in a murine model.

## Introduction

Idiopathic pulmonary fibrosis (IPF) is a devastating disease characterized by the subacute and permanent accumulation of scar tissue in the lung in the absence of an identifiable cause. Currently, available antifibrotic therapies do not reverse established disease, and recent survival estimates are between 2–5 years after diagnosis, which is akin to many aggressive cancers (1). The disease course can be markedly heterogeneous. For example, some patients exhibit only a gradual decline in forced vital capacity (FVC) and may live for many years following diagnosis (2), while others suffer a more progressive clinical course characterized by rapid physiologic decline and death. The molecular mechanisms accounting for these varying phenotypes have yet to be fully defined.

The current pathogenic model of IPF suggests that the progressive decline in lung function is mediated in part by chronic epithelial injury. Epithelial cell death is a common feature of the disease, with areas denuded of epithelium juxtaposed to the characteristic extracellular matrix–producing fibroblastic foci in the lung (3). Moreover, we and others have reported that IPF fibroblasts exhibit an altered and activated phenotype, compared with fibroblasts isolated from normal lung tissue (4). Therefore, understanding the driving forces of epithelial injury and repair, in coordination with aberrant fibroblast activation, is essential to determining the significance of these cellular interactions.

Aberrant angiogenesis is also a prominent histopathological feature of IPF (5). Examination of explanted IPF lung tissue reveals a regional excess of vascular anastomoses juxtaposed to areas of capillary loss (6). An imbalance of angiogenic mediators such as VEGF has also been found in the lungs of IPF patients, where several studies have reported a decrease in lung VEGF protein levels (7–9). However, because other groups have shown VEGF to be unaltered or even elevated in IPF (10, 11), the disease association of this growth factor remains unknown. Apart from its role in stimulating neovascularization, VEGF displays several other functions with direct relevance to the pathogenesis of IPF, such as the stimulation of epithelial proliferation and prevention of epithelial cell apoptosis both in vitro and in vivo (12, 13) and the attenuation of vascular remodeling via protective effects on endothelial cells (14). However, VEGF can also promote the type-2 inflammation that is believed to amplify existing fibrotic responses (15), and therapies targeting VEGF receptor signaling are of modest benefit in the treatment of IPF (16, 17). When viewed in combination, these conflicting data indicate that alterations in the temporospatial expression and/or action of this cytokine might be related to IPF phenotypes and highlight the need for improved modeling systems to study the role of VEGF in mammalian lung fibrosis.

In this study, we determined that IPF patients with high levels of circulating VEGF have preserved lung function and a more benign clinical phenotype. Preclinically, VEGF overexpression in the lung was found to significantly reduce bleomycin-induced mortality and fibrosis via a reduction in epithelial cell death responses. Finally, in vitro, VEGF supported epithelial protection and wound healing via a previously unrecognized mechanism involving endothelial cells.

## Results

### Lung VEGF expression is decreased in IPF.

**Figure 1:**
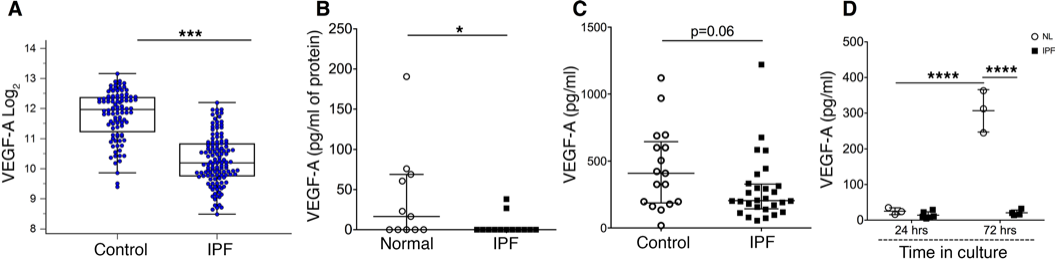
Reduced VEGF in the lungs of IPF patients. (**A**) Reduced levels of lung-tissue *VEGFA* were measured by microarray and compared with Mann-Whitney *U* analysis. (**B**) Luminex-based comparison of VEGF in idiopathic pulmonary fibrosis (IPF) lung tissue (*n* = 13) compared with normal lung tissue taken from the normal nonfibrotic margins of lung tumor resection (*n* = 11). Data were normalized to total mg protein and compared with Mann-Whitney *U* evaluation. (**C**) Decreased bronchoalveolar lavage (BAL) VEGF was measured in IPF (*n* = 28) compared with non-IPF (*n* = 17) BAL, as measured by luminex and compared by Mann-Whitney *U* test. (**D**) Spontaneous release of VEGF from primary normal lung (NL) fibroblasts and IPF lung tissue–derived fibroblasts after 72 hours of culture and compared with ANOVA. In **A–C**, bars represent median and interquartile range, and in **D**, bars represent mean ± SEM. *P < 0.05, ***P ≤ 0.005, ****P ≤ 0.001 or as stated.

In order to begin to characterize the relationship between VEGF and IPF, we evaluated VEGF expression in a gene expression dataset of 219 lung biopsy samples derived from IPF patients (*n* = 123) and normal lung histology samples from control subjects (*n* = 96). This dataset was previously generated by members of our group (18) and is publicly available both in Gene Expression Omnibus (GEO) under accession number GSE47460 and in the Lung Genomics Research Consortium (LGRC) website (http://www.lung-genomics.org/). Subject characteristics are presented in Supplemental Table 1 (supplemental material available online with this article; https://doi.org/10.1172/jci.insight.92192DS1). Comparison of microarray transcript levels of VEGF isoforms and receptors between these groups revealed significant underexpression of *VEGFA* in IPF lung tissue (*U* = 1,242, *Z* = 10.020, *P* < 0.0001, Figure 1A), as well as reduced expression of *VEGFC* (*U* = 1,847, *Z* = 8.42, *P* < 0.0001, Supplemental Figure 1), *VEGFR1* (*U* = 2,094, *Z* = 8.69, *P* < 0.0001, Supplemental Figure 1), and *VEGFR2* (*U* = 2006, *Z* = 8.378, *P* < 0.0001, Supplemental Figure 1). Support of these findings was provided by evaluation of another existing, publicly available expression array dataset from IPF lung biopsies available in GEO under accession number GSE24206 (19). Here, ingenuity pathway analysis revealed that, relative to nonfibrotic control samples, IPF lung demonstrates reduced expression of several VEGF pathway components (*VEGFA*, *VEGFB*, *VEGFC*, *VEGFR1*, and *VEGFR2*) (Supplemental Figure 1). These data show that expression of *VEGFA* and related genes is reduced in the lungs of patients with IPF.

### Lung VEGF protein levels are decreased in IPF.

Having found transcriptional evidence of VEGF deficiency in two independent IPF lung tissue datasets, we validated these findings by evaluating VEGF protein levels in a third set of biopsy samples obtained from the University of Michigan. Results of these studies revealed that, relative to control samples, lung tissue VEGF concentrations were reduced by 87.3% in the IPF tissues (Figure 1B, P** = 0.042). We also compared quantities of soluble VEGF in bronchoalveolar lavage (BAL) fluid from IPF patients and non-IPF controls using Luminex analysis and, interestingly, found only a trend toward reduced BAL VEGF levels in IPF patients that approached but did not reach statistical significance (Figure 1C). These findings suggest that reductions in stromal, but not BAL, VEGF might be mechanistically related to IPF.

### IPF fibroblasts produce low levels of VEGF.

Having shown reduced detection of VEGF in IPF lung tissue, we next sought to understand the potential cellular source of this cytokine. Previous studies of IPF lung tissues have demonstrated a reduced VEGF detection in fibroblasts with sustained expression in alveolar type II cells (AT2, ref. 20), and review of publically available datasets prepared from primary transcriptional evaluation of freshly isolated AT2 cells (GSE94555) and fibroblasts (GSE17978) confirm this finding (21, 22). Interestingly, however, prospective evaluation of VEGF production by IPF fibroblasts has yet to be reported. In order to explore this issue, we assessed primary lung fibroblasts isolated via explant outgrowth from control or IPF lung tissue and used Luminex technology to assess spontaneous VEGF secretion over a 72-hour period. Here, as shown in Figure 1D, relative to supernatants prepared from normal human lung fibroblasts (NHLFs), supernatants obtained from IPF fibroblasts displayed a 93.3% reduction in the spontaneous release of VEGF (*P* = 0.0002). When viewed in combination with the gene expression and protein data shown above, these findings show a relative paucity of VEGF in the IPF lung that is at least partially related to reduced production by fibroblasts.

### Lower plasma VEGF is associated with a progressive IPF phenotype.

**Figure 2:**
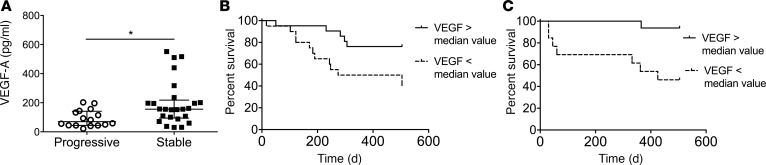
Reduced plasma VEGF is predictive of all-cause mortality in 2 independent IPF cohorts. (**A**) Relative to subjects with stable disease (*n* = 25), idiopathic pulmonary fibrosis (IPF) subjects with progressive disease (*n* = 16) show reduced concentration of VEGF in plasma as compared via Mann Whitney *U* test. Data are shown as median with interquartile range. (**B** and **C**) Kaplan-Meier plot for the composite endpoint of all-cause mortality, absolute drop of > 10% in forced vital capacity (FVC), or acute exacerbation reveals a significant survival benefit for subjects with a VEGF concentration exceeding the median value (top line) relative to those with a plasma VEGF concentration below the median value (bottom line). These findings were derived from a longitudinal cohort of IPF subjects followed at the Yale Interstitial Lung Disease (ILD) Center of Excellence (**B**) and validated in archived samples from the Yale Lung Repository (**C**). *P ≤ 0.05.

The clinical trajectory of IPF can be separated into slowly progressive and rapidly progressive forms (23). In order to determine whether VEGF is related to these clinical phenotypes, we performed ELISA-based quantification of circulating VEGF concentrations in a longitudinal cohort of subjects recruited from the Yale-ILD Center of Excellence. In this cohort, 16 subjects met criteria for having progressive disease (defined as meeting a previously reported composite endpoint of death, acute exacerbation, or an absolute loss of ≥ 10% FVC within 72 weeks of enrollment; ref. 24) and 25 subjects were stable (participants not experiencing these endpoints within the specified time period). Subject characteristics were similar between groups and are shown in Supplemental Table 2. Pairwise comparison of plasma VEGF concentrations between these groups revealed a 54.6% reduction in subjects with rapidly progressive disease (*P* = 0.013, Figure 2A). Importantly, in this cohort, VEGF levels were not associated with clinical factors such as sex, smoking status, age, disease severity as measured by FVC or DLCO, or soluble VEGFR1 or VEGFR2 (Supplemental Figure 2). Next, in order to determine whether a relative reduction in circulating VEGF concentrations might have prognostic significance, we chose the median value of 132.75 pg/ml as a dichotomous variable to stratify subjects into low or high risk for progression. Clinical characteristics of subject in the low or high groups were similar and are shown in Supplemental Table 3. Subsequent Kaplan-Meier analysis of this cohort revealed that values below the median value were significantly associated with all-cause mortality (unadjusted hazard ratio [HR] = 8.809, 95% CI, 2.074–37.42, *P* = 0.0032 Figure 2B). The use of the median value to predict the composite outcome over 72 weeks was then confirmed in an archived validation cohort of 29 subjects from Yale University, where values falling below the median value remained a strong predictor of clinical decline (unadjusted HR =7.39, 95% CI, 1.59–34.95, *P* = 0.0096, Figure 2C). When viewed in conjunction with the tissue data presented above, our findings indicate that a relative underexpression of VEGF in the lung and circulation reflects, and might contribute to, disease progression in IPF.

### Expression of VEGF and its receptors are reduced following bleomycin exposure in mice.

**Figure 3:**
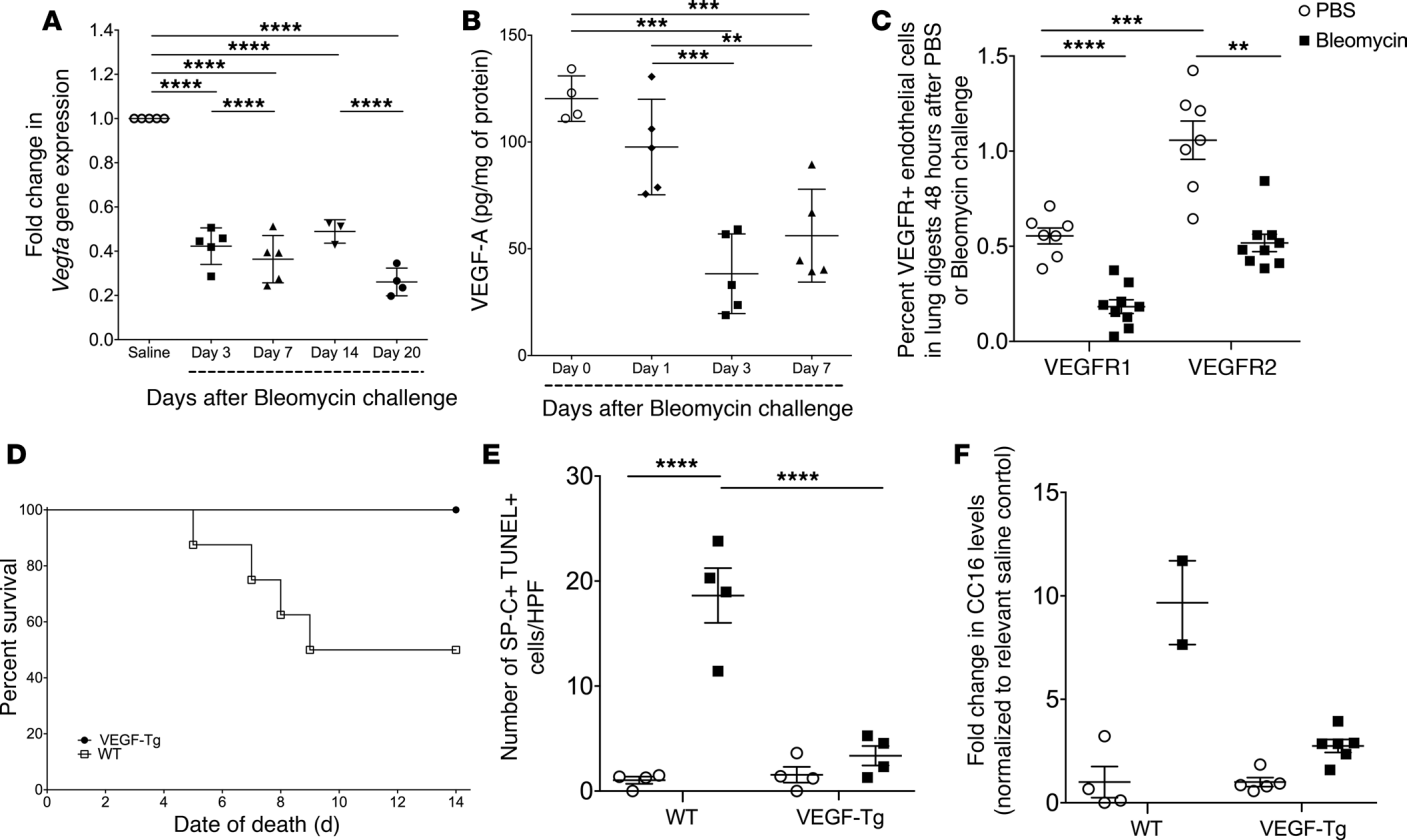
Transgenic *Vegf* overexpression reduced bleomycin-induced lung mortality and epithelial cell damage. (**A** and **B**) Lung tissue *Vegfa* gene expression (**A**) and VEGFA protein levels (**B**) at the indicated time points following intratracheal bleomycin administration in mice. (**C**) Percent of lung endothelial cells that are VEGF receptor 1–positive (VEGFR1-positive) and VEGFR2-positive 48 hours following intratracheal bleomycin measured in lung tissue digests and quantified by flow cytometry. (**D–F**) Kaplan Meier comparison of mortality in bleomycin-challenged WT or VEGF mice (**D**); number of TUNEL^+^ lung epithelial cells identified by surfactant protein C (SP-C^+^) quantified using immunohistochemical analysis (**E**); fold change in bronchoalveolar lavage (BAL) CC16 levels in surviving mice as measured by ELISA (**F**). Bars represent mean ± SEM. **P ≤ 0.01, ***P ≤ 0.005, ****P ≤ 0.001 as stated, two-way ANOVA analysis.

We next sought to correlate our clinical observations with in vivo studies of experimentally induced pulmonary fibrosis. Previous studies of VEGF in this context have yielded conflicting results, with some studies showing fibrosis promoting properties (25) and others demonstrating a beneficial effect (26). In our study, we first characterized VEGF levels in WT mouse lungs following intratracheal bleomycin administration and found significantly reduced expression of this growth factor. Specifically, using quantitative PCR (qPCR), we observed that lung *Vegfa* expression was decreased by approximately 60% at all times examined (Figure 3A, *P* < 0.0001 all timepoints) when compared with control lung expression, a finding that was confirmed at days 3 and 7 by VEGF protein measurements (Figure 3B, P** < 0.001 both comparisons). We also used FACS to quantify the proportion of stromal cells expressing various VEGF receptors where, as expected, we found endothelial cells to be the dominant stromal cell population expressing both VEGFR1 and VEGFR2 in the lungs of unchallenged WT mice (Figure 3C and Supplemental Figure 3). These populations were reduced by 67.5% (*P* < 0.0001) and 49.1% (*P* = 0.008), respectively, 48 hours following the inhalational administration of bleomycin (Figure 3C and Supplemental Figure 3). Detection of VEGR1- or VEGFR2-expressing epithelial cells and fibroblasts was low at baseline (Supplemental Figure 3 and Supplemental Figure 4). In contrast, identification of CD45^+^ hematopoietic cells expressing either VEGR1 or VEGFR2 was high at baseline but unaltered following bleomycin (Supplemental Figure 3 and Supplemental Figure 4). These data show that reduced expression of VEGF and its receptors seen in IPF is recapitulated following bleomycin exposure in mice, further suggesting a relationship with the cellular events leading to fibrosis.

### VEGF overexpression reduces mortality in bleomycin-challenged mice.

Having determined that VEGF expression is reduced in the experimental system most commonly used to study IPF, we thought it likely that VEGF restoration would be of benefit in this model (26). In order to test this hypothesis, we employed a well-characterized transgenic mouse model (VEGF-Tg), in which expression of the human VEGF_165_ gene (from here on referred to as VEGF) is targeted to the lung epithelium under control of a lung-specific, doxycycline-inducible promoter (15). Since these mice are known to have increased VEGF levels in both the BAL and lung parenchyma (15), they afford a unique opportunity to evaluate the potential benefit of VEGF restoration in this model. To this end, age- and sex-matched VEGF-Tg mice or their WT littermates were used to assess the impact of doxycycline-inducible *Vegf* overexpression (15) prior to a single intratracheal exposure of bleomycin. *Vegf* overexpression in the lung results in an acute inflammatory response, which has been shown to modulate vascular remodeling (15). Therefore, we initially induced *VEGF* expression, waited 3 days until the peak inflammatory response subsided, and then challenged the mice with intratracheal bleomycin. The animals received doxycycline in their drinking water continuously following bleomycin administration. Fidelity of transgene expression following bleomycin administration was confirmed by ELISA, where at the 14 day time point, relative to naive animals, BAL fluid of bleomycin-challenged VEGF-Tg^+^ mice displayed stable concentrations of human VEGF165 (Supplemental Figure 5).

We next performed a set of studies in which doxycycline-treated VEGF-Tg mice, or their WT littermates, were randomized to receive 2.5 U/kg of pharmacologic-grade bleomycin and followed for up to 14 days, at which point they were sacrificed and tissue was harvested for fibrosis-relevant endpoints. Here, it was notable that transgenic overexpression of *Vegf* provided a marked survival benefit in bleomycin-challenged mice. Specifically, whereas in the WT mice, greater than 50% mortality was observed with a median survival time of 11.5 days, VEGF-Tg^+^ mice demonstrated no mortality; median survival was 14 days (*P* = 0.05, Figure 3D).

### VEGF overexpression reduces bleomycin-induced epithelial cell death.

The mortality seen in the bleomycin model is typically attributed to the injury and inflammation that follows inhalational delivery of this agent (27). In evaluating these aspects of our model, we found that epithelial cell death responses, as measured by IHC for the epithelial cell–specific marker SPC combined with TUNEL staining, were reduced by 82.2% within 48 hours following bleomycin challenge (*P* = 0.006, Figure 3E and Supplemental Figure 6). As further evidence of a protective effect on lung epithelium, we measured BAL concentrations of CC16, a soluble mediator of epithelial cell injury, in surviving WT and VEGF-Tg mice at the 14-day time point and found a 71.6% reduction in the relative release of this mediator in the VEGF mice (*P* = 0.008. Figure 3F). These data suggest that VEGF overexpression attenuates bleomycin-induced mortality via a cytoprotective effect on the respiratory epithelium.

### VEGF overexpression attenuates collagen accumulation and lung remodeling in the bleomycin model.

**Figure 4:**
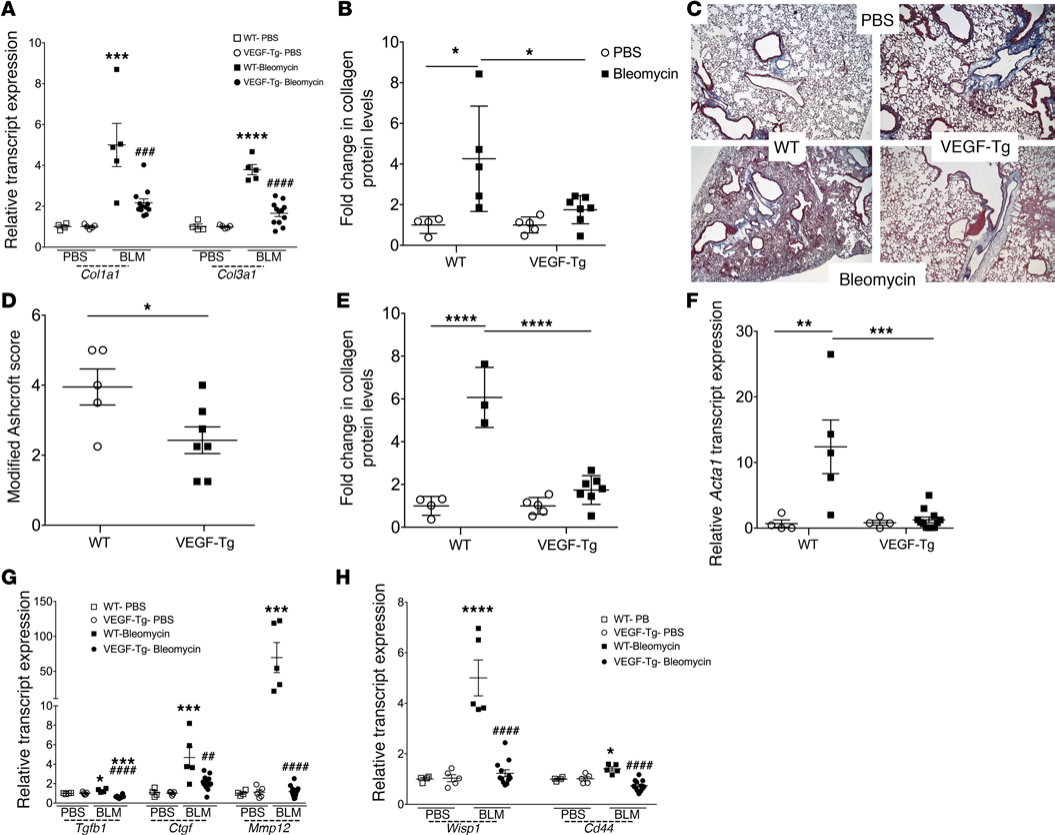
Transgenic *VEGFA* overexpression reduced bleomycin-induced lung fibrosis. *VEGFA* overexpression was induced by doxycycline 3 days before bleomycin administration and was maintained by doxycycline in the drinking water. (**A–D**) Bleomycin-induced collagen gene expression at day 14 as measured by quantitative PCR (qPCR) (**A**), whole lung collagen protein levels at day 14 measured by Sircol assay (**B**), representative lung sections stained with Masson’s trichrome to depict the degree of fibrosis at day 14 in WT and VEGF-Tg mice that received bleomycin or vehicle (PBS) (**C**), and fibrosis semiquantified using modified Ashcroft score analysis (**D**). (**E**) In a separate study, VEGF overexpression was induced for 7 days prior to bleomycin administration and whole lung collagen protein analyzed using the Sircol assay 14 days after bleomycin. (**F–H**) Fold change in whole lung gene expression 14 days after bleomycin as measured by qPCR in *Acta1* (**F**)*; Tgfb1, Ctgf,* and *Mmp12* (**G**); and *Wisp1* and *Cd44* (**H**) gene expression in WT and VEGF-Tg mice that had 3 days of VEGF overexpression prior to bleomycin administration. In the event a gene was undetectable, it was set as zero for this comparison. Bars represent mean ± SEM. *n* = 3–5 WT mice per group, *n* = 7–12 VEGF-Tg mice. *P ≤ 0.05, **P ≤ 0.01, ***P ≤ 0.005, ****P ≤ 0.001 induced by bleomycin in comparison with the relevant strain control, or ^##^*P* ≤ 0.01, ^###^*P* ≤ 0.005, ^####^*P* ≤ 0.001 comparing VEGF-Tg with WT bleomycin challenged mice via two-way ANOVA analysis.

We and others have shown a direct link between epithelial injury events and the magnitude of fibrosis in experimentally induced lung fibrosis (28, 29). Because VEGF overexpression attenuates these aspects of the fibrotic response, we next explored its effect on the collagen accumulation that defines pulmonary fibrosis. In order to have sufficient mice for analysis, the bleomycin dose was reduced to 1.25 U/kg. Here, we evaluated relative expression of *Col1a1* and *Col3a1* by qPCR and found a 56.5% reduction in *Col1a1* and a 56.2% reduction in *Col3a1* in the VEGF-exposed lung (*P* < 0.0001 both comparisons, Figure 4A). These transcriptional measurements were complemented by biochemical quantification of collagen, which is widely accepted as the gold-standard endpoint for studies of lung fibrosis (30) (here measured by the Sircol assay), where whole lung samples from VEGF-Tg mice exhibited a 40.1% reduction in the degree of collagen accumulation (*P* = 0.0073, Figure 4B). These effects on collagen accumulation were accompanied by a reduction in the histologic appearance of lung remodeling based on trichrome stains and modified Ashcroft Scores (*P* = 0.0352; Figure 4, C and D). To ensure that the reduced fibrosis was not due to the timing of transgene activation, we assessed the effects of bleomycin administration after 7 days of *VEGF* overexpression and observed a similar reduction in the fold change in collagen levels (*P* = 0.00393, Figure 4E). These data show that exposure to VEGF during the initial and intermediate phases of fibrogenesis attenuates many of the well-accepted endpoints used to quantify fibrosis in the bleomycin model.

### VEGF overexpression reduces the expression of multiple fibrosis associated genes.

In order to determine whether VEGF overexpression affects additional aspects of the fibrotic response, we used qPCR to evaluate transcripts associated with myofibroblast activation, TGFβ1 production, and Wnt signaling. Results of these studies revealed that VEGF overexpression led to significant reductions in the myofibroblast-associated marker *Acta1* (*P* < 0.0001, Figure 4F), *Tgfb* (*P* < 0.0001, Figure 4G) and its associated regulators *Ctgf* and *Mmp12* (*P* < 0.001 and *P* < 0.0001, respectively, Figure 4G), and markers of Wnt pathway activation *Wisp-1* and *Cd44* (31) (*P* < 0.0001 both comparisons, Figure 4H). When viewed in combination, these data demonstrate that VEGF overexpression suppresses a number of profibrotic mechanisms related to myofibroblast activation, TGFβ expression, and Wnt pathway activation.

### VEGF does not directly influence injury in cultured human epithelial cells.

**Figure 5:**
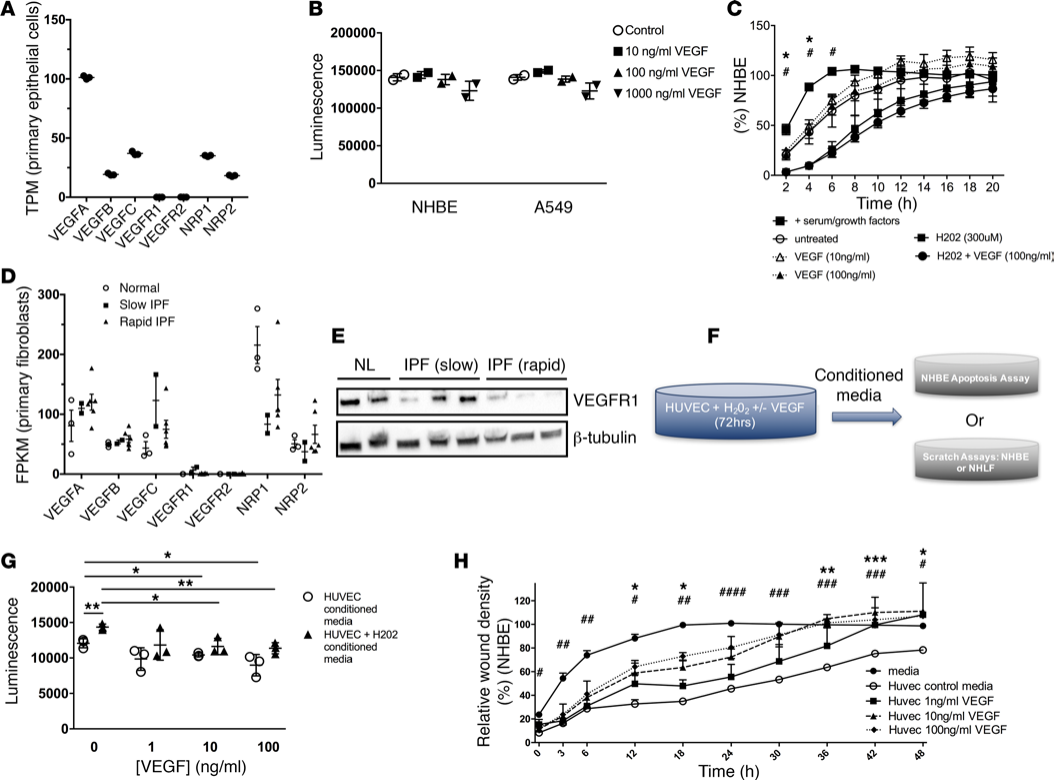
Protective effect of VEGF on epithelial cells occurs indirectly through its effect on endothelial cells. (**A**) Primary lung epithelial cells were assessed by RNA sequencing analysis for VEGF receptor expression (*n* = 3). (**B**) Effect of VEGF (0–1,000 ng/ml) on lung epithelial cell (normal human bronchial epithelial cells [NHBE] or A549) caspase 3/7 release after 24 hours of in vitro stimulation. (**C**) Effect of H_2_O_2_ in the presence of absence of VEGF on epithelial wound closure. (**D** and **E**) Normal or idiopathic pulmonary fibrosis (IPF) lung fibroblast cultures were stained with fluorescent conjugated anti-SSEA4 (stage-specific embryonic antigen 4) antibodies. SSEA4-negative lung fibroblasts were sorted from SSEA4-positive progenitors; RNA was extracted from the sorted cells and subject to RNA sequencing analysis. The resulting RNA-Seq reads were aligned to the human genome build hg18 and normalized fragments per kilobase of transcript per million mapped reads (FPKM) values pertaining to VEGF and VEGF receptor transcripts were mined. (**D**) Depicted is the average FPKM values from 3 normal, 2 slow progressing, and 5 rapid progressing IPF lung SSEA4-negative fibroblasts. (**E**) Representative Western blot of VEGFR1 expression in primary stromal cultures from nonfibrotic lungs (NL) (*n* = 2) and IPF lungs with rapid and slow progressing disease (*n* = 3). (**F**) Schematic showing the generation of HUVEC conditioned media. (**G**) HUVEC were incubated with or without H_2_0_2_ and different concentrations of VEGF (0–100 ng/ml), to elicit HUVEC-conditioned media. This was then added to NHBE cells, and apoptosis was measured by caspase 3/7 activity after 48 hours of incubation. (**H**) Relative wound healing percentage on NHBE cells cultured in either their own media or HUVEC-conditioned media (without or with added VEGF). Data is representative of 3 separate experiments. *P ≤ 0.05, **P ≤ 0.01, ***P ≤ 0.005 as stated or HUVEC-conditioned media compared with 100ng/ml condition media; ^#^*P* ≤ 0.05, ^##^*P* ≤ 0.01, ^###^*P* ≤ 0.005, ^####^*P* ≤ 0.001 HUVEC-condition media compared with control media. All analysis were performed via one-way ANOVA analyses with Bonferroni significance post-testing.

Because the studies outlined above revealed that VEGF overexpression resulted in a cytoprotective benefit on AT2, we employed to ex vivo modeling to better understand how these entities might interact. Primary human AT2 cells have been reported to express very low levels of VEGFR1 and VEGFR2, and analysis of currently available AT2 profiling reveals similar observations in IPF cells (GSE94555, ref. 22). Because primary AT2 cells are difficult to obtain and not commercially available, we used two human lung epithelial cell lines, the A549 cell line and normal human bronchial epithelial cells (NHBEs) to determine whether the reduction of epithelial injury observed in vivo was mediated through a direct effect of VEGF. The A549 cell line was used as a model of human AT2 cells displaying low-level expression of VEGFR1 and VEGFR2 (32). In contrast, when NHBE were subject to RNA-Seq, they were found to be positive for *VEGFA*, *VEGFB*, *VEGFC*, and the VEGF coreceptors *NRP1* and *NRP2*, but they were negative for *VEGFR1* and *VEGFR2* (Figure 5A). Therefore, NHBEs were used as an example of primary lung epithelium that lacks either receptor.

Next, in an attempt to determine whether administration of VEGF was sufficient to attenuate epithelial cell injury ex vivo, we exposed A549 and NHBE cells to serum starvation and attempted to “rescue” injury with increasing concentrations of VEGF. Here, we found that even high doses of VEGF (100 ng) delivered for up to 24 hours failed to attenuate injury, as measured by cleaved caspase 3 levels in either cell line (Figure 5B). Because epithelial cell injury and regeneration appear to be closely linked in vivo (33, 34), we also assessed the impact of VEGF on epithelial wound closure and found that administration of this growth factor failed to support NHBE wound closure in a scratch assay (Figure 5C). Bleomycin elicits epithelial cell apoptosis via an increase in ROS (35), and IPF is associated with a ROS imbalance in the lung (36). In our studies, hydrogen peroxide (H_2_0_2_) delayed wound closure, yet VEGF did not affect this delay (Figure 5C). When viewed in combination, these data suggest that if VEGF influences epithelial homeostasis, the benefit may be mediated through a non–cell autonomous mechanism.

### Fibroblasts are not a major source of VEGFR2 expression.

Emerging evidence indicates an important role for fibroblasts in the maintenance of alveolar epithelial integrity in many settings (37). In order to assess whether the in vivo effects of VEGF might occur via epithelial/fibroblast interactions, we profiled the expression of the VEGFRs in primary stromal cell cultures derived from the lungs of patients with stable or progressive IPF and nonfibrotic lungs (NL). Progenitor cell populations were first removed by FACS sorting on the basis of SSEA4 expression (38), and the subsequent stromal cells were submitted to RNA-Seq analysis. These studies revealed that revealed that fibroblast cells do not express *VEGFR2* but present low expression of *VEGFR1* (Figure 5D), which was confirmed at protein level by Western blot (Figure 5E). The expression of the VEGF family receptors *NRP1* and *NRP2* was also seen on primary fibroblasts (Figure 5D). Since VEGFR2 is the major receptor for VEGFA, these data suggest that the cytoprotective function of VEGF is unlikely to occur directly through fibroblasts.

### VEGF’s protective effects on epithelial injury are mediated via endothelium.

Endothelial cells are VEGFR2-positive (39), and our evaluation of the uninjured mouse reveals a substantial portion of stromal cells to be endothelial cells expressing VEGFR2. Recent studies have proposed the presence of an endothelial niche that contributes to epithelial integrity in several models of lung injury, including pulmonary fibrosis (40, 41). In order to determine whether epithelial interactions with activated endothelial cells might explain our findings, we cultured endothelial cells (HUVEC) in the presence or absence of H_2_0_2_ to promote endothelial cell activation, and in the presence or absence of VEGF to generate conditioned media (Figure 5F). We then took this conditioned media and applied it to separately cultured, serum-starved NHBEs to assess whether mediators derived from endothelial cells could alter epithelial cell injury. We found that conditioned media from H_2_0_2_-treated endothelial cells increased epithelial cell apoptosis compared with non-H_2_0_2_–conditioned media, as measured by elevated caspase 3/7 activity (Figure 5G), in a manner that was reversed by HUVEC pretreatment with VEGF (*P* ≤ 0.05 all comparisons, Figure 5G). Similar findings were seen in studies of epithelial repair, where addition of VEGF-treated HUVEC conditioned media improved wound closure in NHBEs (*P* ≤ 0.01 all comparisons, Figure 5H and Supplemental Figure 7). These results support a role for VEGF in protecting the epithelium via a non–cell autonomous mechanism involving the endothelium.

### VEGF’s protective effects are associated with enhanced production of TSP1.

**Figure 6:**
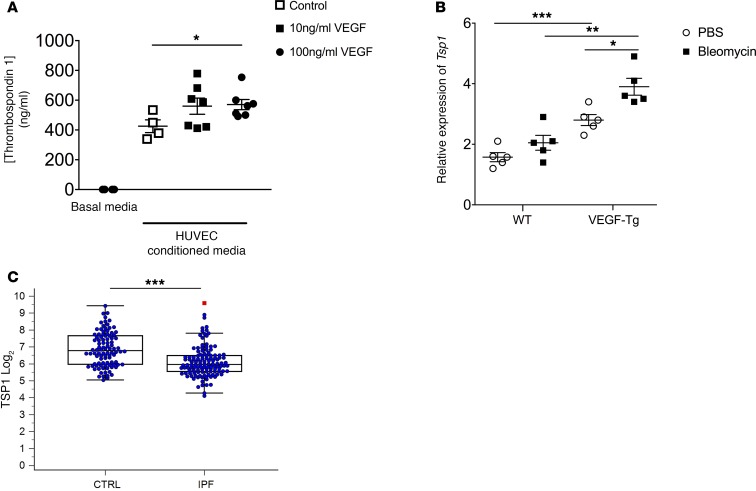
VEGF enhances thrombospondin-1 in vitro and in vivo, and expression of this gene is reduced in IPF. (**A**) Relative to media obtained from unstimulated HUVEC (open squares), media obtained from HUVEC stimulated with 100 ng/ml show a significant increase in thrombospondin 1 (TSP1) concentrations. (**B**) Relative to bleomycin-challenged WT mice, lungs obtained from VEGF-transgenic (VEGF-Tg) mice 48 hours after intratracheal bleomycin demonstrate increased whole lung gene expression of *Tsp1*. (**C**) Relative to nonfibrotic control tissues, idiopathic pulmonary fibrosis (IPF) lung tissues demonstrate significantly reduced expression of *TSP1* in an existing microarray dataset. Bars in **A** and **B** represent mean ± SEM; bars in **C** represent median and interquartile range. *P ≤ 0.05, **P ≤ 0.01, ***P ≤ 0.005. One-way ANOVA analysis for **A** and **B**. Mann-Whitney *U* evaluation for **C**.

Finally, we sought to determine the potential mechanism through which the endothelium might contribute to epithelial homeostasis. Endothelial cells have been shown to support alveolar epithelial repair via the regulated secretion of thrombospondin-1 (TSP1) (41), a secreted glycoprotein that regulates cell-to-cell communication and matrix interactions (41). Interestingly, absence or underexpression of *Tsp1* in mice causes a lung phenotype characterized by acute injury, and organizing pneumonia (42, 43) and *Tsp1* deficiency impedes alveolar epithelial repair in both the bleomycin model and in ex vivo studies (41, 44). Thus, we thought it possible that the support of epithelial cell homeostasis by VEGF stimulated HUVECs might involve TSP1. This prediction proved correct, as conditioned media from VEGF-treated HUVECs revealed a dose-dependent increase in TSP1 concentrations that reached statistical significance at the 100 ng/ml dose (*P* = 0.028, Figure 6A). These findings were highly specific to TSP1, as additional mediators such as IL25, IL33, TSLP, IFNγ, IL10, IL4, or IL13 were undetectable in the HUVEC media in the presence or absence of VEGF treatment (data not shown). As a proof-of-principle exploration of the VEGF-TSP1 axis in mammalian lung fibrosis, we measured *Tsp1* transcripts in the lungs of bleomycin-treated, WT, and VEGF-Tg mice and found a 1.7-fold increase in the relative expression in the VEGF-exposed lungs at the 48-hour time point following bleomycin (*P* = 0.0011, Figure 6B). In a human correlate of these observations, we evaluated the LGRC dataset, where we found a 12% reduction in median *TSP1* expression relative to nonfibrotic controls (*U* = 3339; *Z* = 5.513; *P* < 0.0001, Figure 6C), a finding that was confirmed in the second dataset (GSE24206) where a 0.52-fold reduction in TSP1 was observed in samples from the IPF lung (*P* < 0.0001, data not shown). When viewed in combination, these data suggest that the cytoprotective effects of VEGF are mediated via a mechanism involving TSP1 and that this axis is deregulated both in experimentally induced lung fibrosis and in the setting of human disease.

## Discussion

These findings lend new insight into the complex role that VEGF might play in pulmonary fibrosis. Specifically, we show that VEGF expression is reduced in the lungs and blood of patients with IPF, and in the latter compartment, low VEGF displays an association with poor clinical outcomes. In preclinical studies using the bleomycin mouse model, we found reduced expression of VEGF and its receptors in the lung. Lung-targeted overexpression of *Vegf* markedly attenuates bleomycin-induced epithelial cell death responses, mortality, collagen accumulation, and histologic evidence of tissue remodeling. In vitro, VEGF treatment confers a protective effect on epithelial cell apoptosis via an indirect endothelial cell–dependent mechanism involving TSP1. Collectively, our data indicate that a reduction of VEGF in IPF may contribute to the ongoing injury that is thought to promote fibroproliferation and disease progression.

The relationship of VEGF to IPF remains a controversial area. Several studies have reported lower levels of this agent in BAL (8, 45) and lung (9) samples from IPF patients, while other studies report the opposite (10, 11). In our study, expression of VEGF and its receptors was reduced in IPF lung tissue, and VEGF levels were lower in the circulation of IPF subjects with more progressive disease. These findings suggest two competing hypotheses: either VEGF preserves lung function or preserved lung function contributes to higher VEGF levels. The former interpretation is supported by the current study and other work showing a protective role for VEGF in the bleomycin model (26), whereas the latter interpretation is supported by studies demonstrating epithelium and endothelium (both of which are obliterated by fibrosis) to be a dominant source of VEGF in the lungs of patients with IPF (20). Our study also suggests that interventions that increase VEGF might be efficacious in IPF, though because VEGF-receptor inhibition has been shown to be safe and efficacious in the treatment of IPF (16, 17), the role of VEGF as it relates to its receptors and coreceptors in the IPF disease state will require further study.

Our studies also reveal a previously unrecognized role for transgenic VEGF overexpression in suppressing bleomycin-induced injury, mortality, and fibrosis. While one prior study showed that targeted deletion of VEGF in myeloid cells worsened bleomycin-induced fibrosis and remodeling (26), to our knowledge, ours is the first study to demonstrate a direct benefit for VEGF administration in this model. Because VEGF is a proangiogenic mediator and inhibition of angiogenesis-associated pathways has been reported to attenuate bleomycin-induced lung fibrosis (46–48), our findings might be somewhat surprising. However, beyond its angiogenic properties, VEGF also exerts epithelial-protective functions in the lung (13, 49), and transgenic VEGF expression has been shown to protect mice against hyperoxia-induced lung injury (50). In addition to direct cytoprotection, VEGF has been shown to mediate efferocytosis in several settings (49, 51, 52), which would be expected to attenuate inflammation and shift the lung microenvironment toward favoring repair. Furthermore, a prorepair association of VEGF in the lung has also been observed in adult respiratory distress syndrome (ARDS), where BAL obtained from recovering ARDS patients showed increasing concentrations of VEGF (53). Thus, while our study does not specifically explore all aspects of epithelial homeostasis in vivo, our data do suggest that the effect of VEGF on fibrosis and remodeling are mediated at least partially through beneficial effects on the alveolar epithelial cell death responses that are believed to drive fibrosis in the mammalian lung.

One particularly fascinating aspect of our study is the delineation of, to our knowledge, a highly novel interaction between VEGF, endothelial cells, and epithelial integrity in the setting of experimentally induced lung fibrosis. Our animal modeling indicates that transgenic overexpression of VEGF exerts important protective effects on the alveolar epithelium, and our ex vivo studies suggest that the epithelial-protective functions of VEGF occur in a non–cell autonomous function mediated by the endothelium. While we have not specifically demonstrated this finding in vivo, our finding that lung endothelial cells express high levels of VEGFR2, the major VEGF receptor, suggests that our observations in the VEGF model are mediated in part via this population of cells. These observations add to the emerging recognition that endothelial cells form a niche for epithelial cells that both protects cells from injury and promotes repair via a number of mechanisms (40, 41, 54). It should also be mentioned that, while our data convincingly show that VEGF-stimulated endothelial cells are important in our modeling system, we have not ruled out the effect of VEGF signaling in other cells — such as macrophages, epithelial cells, and pericytes — which will require the generation of mice with cell-specific deletion of VEGFRs.

Another aspect of our study is the identification of a potential role for TSP1 in these processes, which is consistent with prior studies in humans, in which TSP1 levels were reported to be reduced in BAL fluid obtained from subjects with IPF (55). Our work is also supported by several studies in mice, in which TSP1 deficiency causes a baseline phenotype characterized by spontaneous alveolar damage that is worsened by exposure to inhaled bleomycin (42, 44), and treatment with a TSP1 mimetic attenuates inflammation and fibrosis in the bleomycin model (56). In the context of these prior studies, our data suggest that VEGF-stimulated endothelium might regulate epithelial homeostasis via a mechanism involving TSP1. When viewed in this light, it is significant that one recent study showed that TLR4 expression by lung endothelium was both sufficient and necessary to protect the alveolar epithelium from oxidant-mediated injury caused by exposure to hyperoxia (57). Given the emerging role of TLR4 in mediating epithelial injury and repair responses (58), because TSP1 serves as a danger-associated signal for TLR4 (59), our data suggest an intriguing relationship between endothelial VEGF responses, TSP1, innate immunity, and epithelial homeostasis that will require further exploration.

While important, our study has several limitations. We have not determined the reason VEGF levels are low in the IPF lung and circulation, though our human data suggest that reduced expression by fibroblasts is likely to play a role. We have not determined the precise relationship between VEGF and disease progression, which will require access to larger cohorts with more extensive clinical followup. We have not determined the cells through which VEGF exerts its cytoprotective benefit in the bleomycin model, which will require the generation of mice with cell-specific deletion of VEGF receptors, and we have not evaluated whether VEGF overexpression shows a similar benefit when administered in a therapeutic schedule. Lastly, we have not explicitly shown that TSP1 is protective, which will require additional studies using ex vivo culture systems and additional murine modeling.

In conclusion, these findings support the contention that VEGF protects the respiratory epithelium from exogenous injury via a non–cell autonomous mechanism involving the endothelium. Given the emergence of interventions targeting VEGF receptors in IPF and cancer, further assessment of these findings is warranted.

## Methods

### Subject recruitment.

Individuals with a diagnosis of IPF according to the 2002 ERS/ATS consensus statement (1) were recruited for involvement in these studies between 2006 and 2011, and a separate cohort of patients recruited according to the revised 2011 criteria (23) was recruited between 2011 and 2016 (from the Yale ILD Center in New Haven, Connecticut, USA). Participants were treatment naive at the time of blood draw, treated with a variety of treatment regimens, and followed closely with physiological studies and capture of clinical information during acute events. Using methodology that has been validated for measuring disease progression during the 72 weeks of followup, a composite of all-cause mortality, absolute decrease in percent predicted FVC of >10% (60), or acute exacerbations of IPF were defined according to the criteria of Collard et al. (61). Age-matched normal controls were recruited from the local community. For lung biopsy tissue, IPF samples were taken at time of diagnosis from IPF patients. The nonfibrotic, normal margins of lung tumor resections were used for normal control tissue.

### Bleomycin model of pulmonary fibrosis.

To induce pulmonary fibrosis, male and female C57BL/6 (WT) mice or VEGF-Tg mice received an intratracheal bleomycin challenge (Blenoxane, Teva Pharmaceuticals) as previously described (46, 62). The CC10-tTS-rtTA-VEGF-Tg mice used in this study have been described previously (15). These mice use the Clara cell 10-kD protein (CC10) promoter to specifically target the human *VEGF165* gene to the lung. These mice were backcrossed for more than 10 generations onto the C57BL/6 background, bred, and genotyped as previously described (15).

### Flow cytometry of digested lung tissue.

FACS-based enumeration of VEGF receptor expression was performed on lung tissues that had been digested and processed, as we have previously described (63). Antibodies used were CD45 (45-0451, BD Pharmingen), EpCAM (11-5791, eBioscience), PECAM (11-0311, eBioscience), COLIA1 (600-401-103-0.5, Rockland), VEGFR1 (FAB4711A, R&D Systems), and VEGFR2 (12-5821, eBioscience).

### CC16 assay in BAL.

CC16 concentrations were evaluated in BAL specimens using a commercially available ELISA based assay (RayBiotech) according to manufacturer instructions.

### Sircol assay.

Soluble collagen content was quantified using the Sircol assay (Biocolor) according to the manufacturer’s instructions, as we have previously described (64).

### Modified Ashcroft Scores.

The extent of lung remodeling was quantified using a minor modification of the Modified Ashcroft Scoring system (65), as we have previously described (64).

### In vitro epithelial and endothelial assays.

HUVEC cells were supplied by Lonza and maintained in EGM-2 Bulletkit Biowhittaker (Lonza). To harvest conditioned media, HUVECs were cultured for 24 and 48 hours in media, 10% serum, and 0, 1, 10 or 100 ng/ml of recombinant VEGF (R&D systems). Media was collected at these time points. NHBE (nonsmoker, donor 105116) maintained in BEGM media (Lonza) were used for epithelial cell apoptosis and wound-healing assays. NHBE were cultured for 24 hours and 48 hours, respectively, in either their normal culture media, increasing concentrations of VEGF_165_, H_2_O_2_, or HUVEC-conditioned media, previously filtered through a 40-μm cell strainer.

### In vitro epithelial, fibroblast, and endothelial assays.

Human umbilical vein endothelial cells (HUVEC, Lonza) were cultured for 48 hours in media, 10% serum, and 0, 1, 10, or 100 ng/ml of recombinant human VEGF_165_ (R&D systems) to resultant conditioned media filtered prior to use to ensure the media is free of cells. NHBE (nonsmoker, donor 105116) were cultured in ImageLock plates (Essen BioScience) overnight until they reached confluency. Cells were then scratched 3 times using pipette tips (Eppendorf), washed with PBS, and then incubated in HUVEC-conditioned media or relevant control media for 48 hours at 37^o^C, 5%CO_2_. Wound closure was measured as percentage of closure and quantitated via Incucyte technology (Essen Bioscience). For epithelial cell apoptosis, NHBE were cultured for 24 hours in either normal culture media or in HUVEC-conditioned media, and apoptosis was quantitated using Caspase-Glo^R^ 3/7 Assay substrate (Promega), following the manufacturer’s protocol.

### Flow cytometry of primary lung fibroblasts.

Lung fibroblasts were trypsinized, washed with PBS, and resuspended at a concentration of approximately 1 x 10^7^ cells/ml in flow cytometry staining buffer (DPBS + 1% BSA + 0.02% NaN_3_). Approximately 1 × 10^6^ cells in a volume of 100 μl were blocked for 15 minutes on ice using 2 μg of human IgG (R&D systems). Fluorescent conjugated or isotype control antibodies were added to the cells at a dilution of 1:50. The cells were incubated with the anti-CD90 and anti-SSEA4 antibodies (BioLegend) for 15 minutes on ice in the dark, subsequently washed twice with flow buffer, fixed in 3% neutral buffered formalin (NBF), and subsequently analyzed by flow cytometry using the MASCQuant Analyzer 10 (Miltenyi Biotec) flow cytometer and FlowJo software (Tree Star Inc.) for analysis.

### Protein and mRNA analyses.

For protein analysis in lung tissue, lung biopsy tissue from IPF/UIP patients or from the normal margins of lung tumor resections, or lungs from mice, were homogenized in antiprotease buffer (Roche Diagnostics) and processed as previously described (46). BAL was taken from the mice by flushing the lungs with sterile PBS, while serum was prepared from a postmortem blood draw. Whole lung lobes were dissected for histological and biochemical analysis. Cytokine levels were measured by bead based LuminexÔ analysis or specific ELISA (R&D Systems). In some instances, mediator levels were normalized to protein content, which was determined using a standard Bradford protein assay. For gene analysis, total RNA was obtained using TRIzol reagent (Invitrogen), according to the manufacturer’s instructions. Gene expression levels were quantitated using real time RT-PCR (Applied Biosystems), according to the manufacturer’s protocols. Undetectable expression was set as zero for comparisons. Transcript levels of genes of interest were normalized to β-actin or 18S mRNA.

### Histologic analysis.

Formalin-fixed and paraffin-embedded lung sections were stained for TUNEL (Roche Diagnostics) and Surfactant Protein C (Santa Cruz Biotechnology Inc., sc-13979) or with Masson’s trichrome stains to visualize collagen deposition as per manufacturer’s instructions.

### RNA-Seq analysis.

Normal and IPF stromal cultures were expanded, trypsinized, and stained with anti-SSEA4 antibody (BioLegend, clone MC-813-70, catalog 330408). SSEA4^+^ (2 normal, 4 slow IPF, and 5 rapid IPF) and SSEA4^–^ (3 normal, 2 slow IPF, and 6 rapid IPF) cells were sorted (between passages 6 and 9) using a BD FACSAria III and subsequently spun down and resuspended in QIAzol lysis reagent (Qiagen) for RNA extraction. A minimum of 700 ng of total RNA was used in Dynabeads mRNA DIRECT Micro Purification Kit (Ambion). The libraries were prepared using the Ion Total RNA-Seq Kit v2 (Life Technologies). Samples were then loaded into an Ion Torrent for amplification onto Ion Sphere Particles using Ion PI Template OT2 200 Kit v3 (Life Technologies), Ion PI Sequencing 200 Kit v3 (Life Technologies) for sequencing chemistry, and Ion PI Chip Kit v2 (Life Technologies) sequencing ChIP. Samples were sequenced to at least 10 million reads. Raw reads in FASTQ format were aligned to the UCSC human reference genome (hg19) using TopHat (https://ccb.jhu.edu/software/tophat/index.shtml). Gene expression was calculated using a gene transfer file (GTF) from UCSC genes and normalized read quantification as fragments per kilobase of transcript per million fragments mapped (FPKM) calculated with Cufflinks. FPKM values for known VEGF receptors and various transcripts, enriched in SSEA4^+^ cells relative to SSEA4^–^ cells, were graphed and depicted.

### Evaluation of gene expression data.

To evaluate the differences in expression levels between candidate genes, we used the Mann-Whitney *U* test for independent samples of the normalized, log_2_-transformed microarray expression values. Statistical significance was defined as *P* < 0.05. For the microarray studies of IPF lung tissue, deidentified tissues collected by the LGRC were employed (18)

### Statistics.

Normally distributed data were expressed as means ± SEM and assessed for significance by Student’s *t* test (two-tailed) or ANOVA as appropriate. Nonparametric data were expressed as median and interquartile range and compared using Mann-Whitney *U* test. Subject demographics were compared using Student’s *t* test or Mann Whitney analysis. Categorical variables were compared using Fisher’s exact test. *P* values were determined for multiple comparisons using the Bonferroni correction. Values of *P* ≤ 0.05 (* or ^#^), *P* ≤ 0.01 (** or ^##^), *P* ≤ 0.005 (*** or ^###^), and *P* ≤ 0.001 (**** or ^####^) were considered significant.

### Study approval.

All human studies were performed with human institutional care approval at Yale University School of Medicine, the University of Michigan School of Medicine, and Hôpital Bichat. All individuals provided informed consent prior to their participation in the study. All in vivo bleomycin studies were conducted according to UK Home Office regulations and approved protocols or according to Yale School of Medicine IACUC, in accordance with federal guidelines.

## Author contributions

LAM, CMH, and ELH conceived and designed the study and wrote the manuscript. MH, DMH, AC, HS, YZ, ALC, and XP performed in vitro and/or in vivo studies and/or conducted human sample analyses. MAS, TM, and DAK participated in study design and data analysis. BC provided IPF BAL samples and analyses. ADOA and MWM performed evaluation of human plasma specimens. JAE and CGL provided the VEGF-Tg mice. CR assisted with data analysis and statistical evaluation. MG assisted with subject recruitment and data management. JDHM performed and analyzed microarray data. BH analyzed microarray data.

## Supplementary Material

Supplemental data

## Version 1 08/14/2017

Electronic publication
