# Supplementary material for: Antifibrotic role of vascular endothelial growth factor in pulmonary fibrosis
Source: JCI Insight. 2017 Aug 17;2(16):e92192. doi: 10.1172/jci.insight.92192 (PMC5621899; doi:10.1172/jci.insight.92192)
Supplement: Supplemental data [file jciinsight-2-92192-s001.pdf]

Supplemental Figures

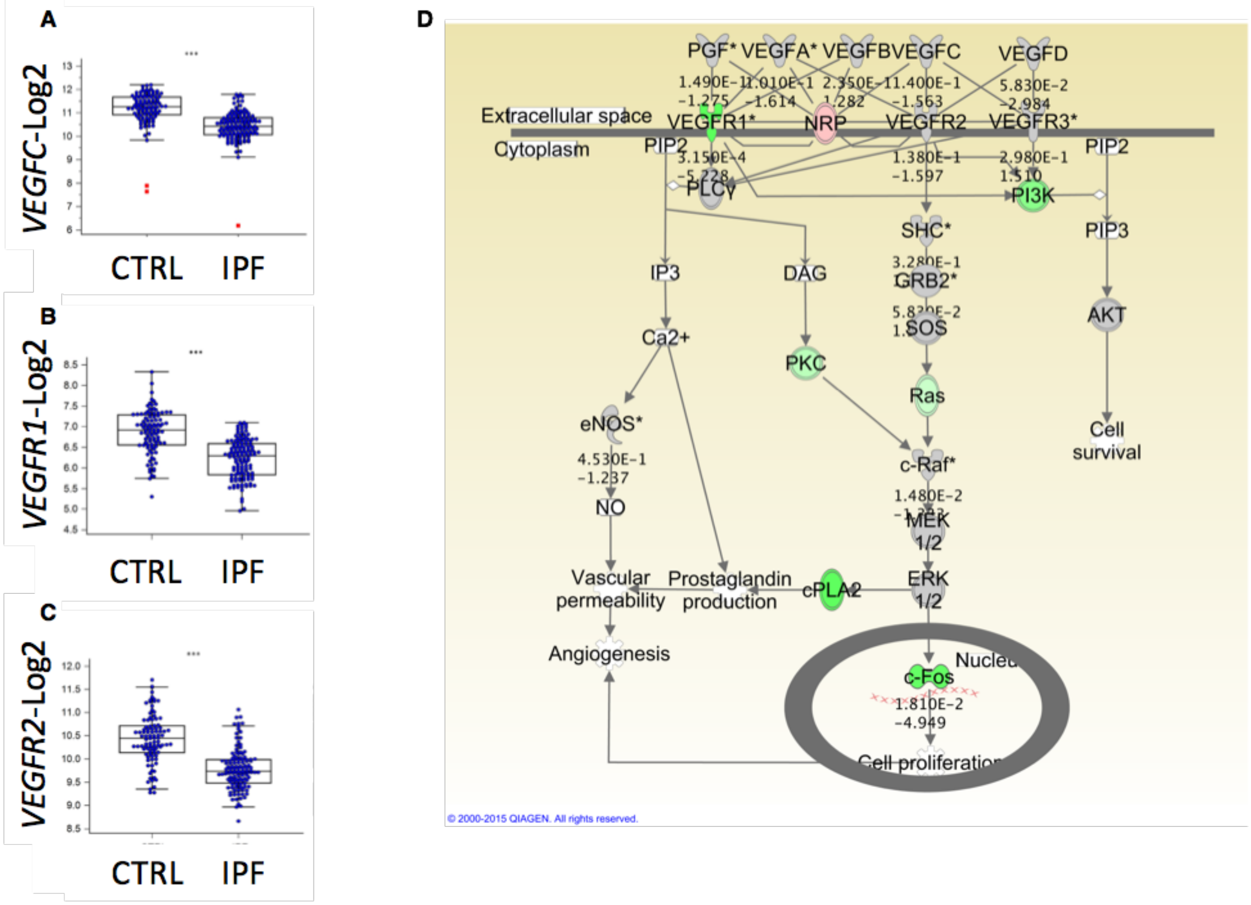

Supplemental figure 1. A-C. Microarray based profiling of the LGRC dataset reveals that relative to control lung tissue (N=96), IPF lung tissue (N=123) shows reduced expression of *VEGFC* (A), *VEGFR1* (B) and *VEGFR2* (C). These comparisons were all made via Mann-Whitney test. D. Six normal, 8 IPF lung biopsies and 9 IPF lung explants derived gene expression arrays (GSE24206) were analyzed using Geo2R. Briefly, groups were defined as: IPF lung biopsy (early IPF) vs normal or IPF lung explant (late IPF) vs normal. P-values were calculated and adjusted using Benjamini & Hochberg (FDR). The resulting fold changes and P-values were uploaded onto ingenuity IPA. Ingenuity was set to only consider transcripts with a fold change of ≥1.5 and a p-value of ≤0.05. The resulting data was overlaid to Ingenuity's VEGF canonical pathway. Shown are the expression fold change in IPF relative to normal (top value) and the p-value across the samples (bottom value) following Student's t test analyses.

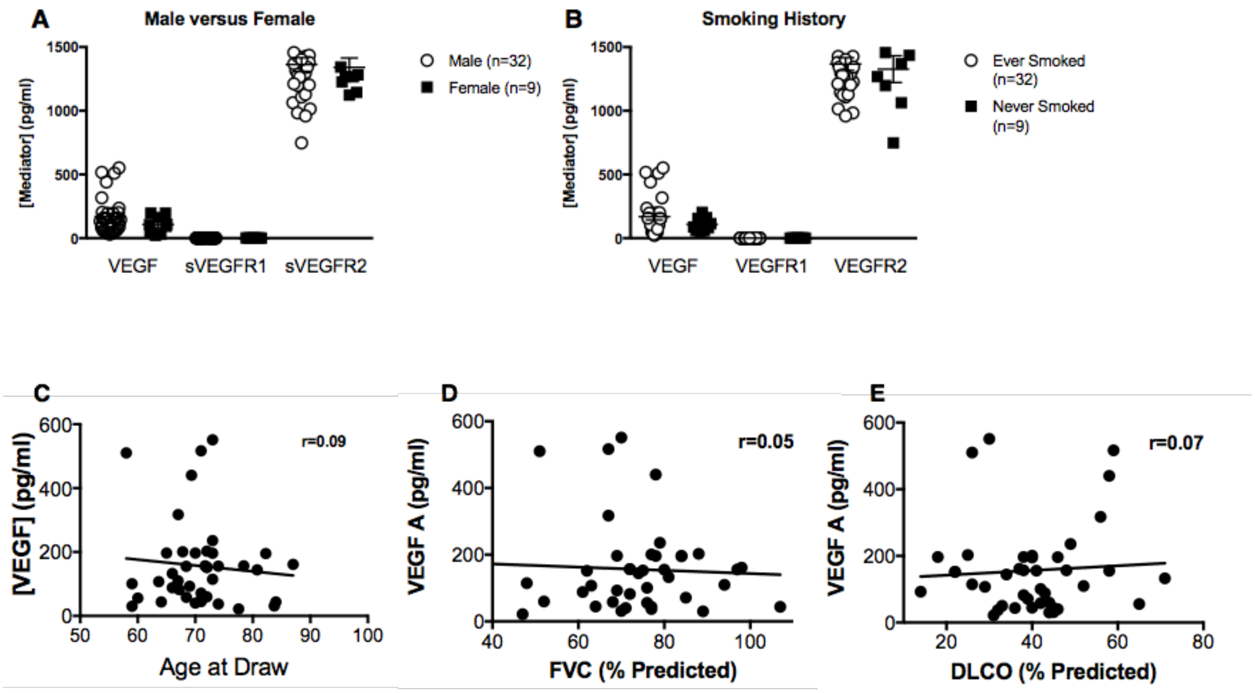

Supplemental figure 2. Correlations of circulating VEGF, soluble VEGFR1 and soluble VEGFR2 with sex, smoking history and age in IPF. Plasma VEGF, soluble VEGFR1 and soluble VEGFR2 levels in IPF patients (n=41) were separated based on male (n=32) or female (n=9) basis and compared (A), comparisons on smoking history (B) were also conducted. Bars represent the mean ± S.E.M. The correlations between plasma VEGF levels and age of IPF patient (C), FVC percent predicted (D) and DLCO percent predicted (E). Each data point is an individual patient (n = 41). No statistical difference was observed in any of the comparisons.

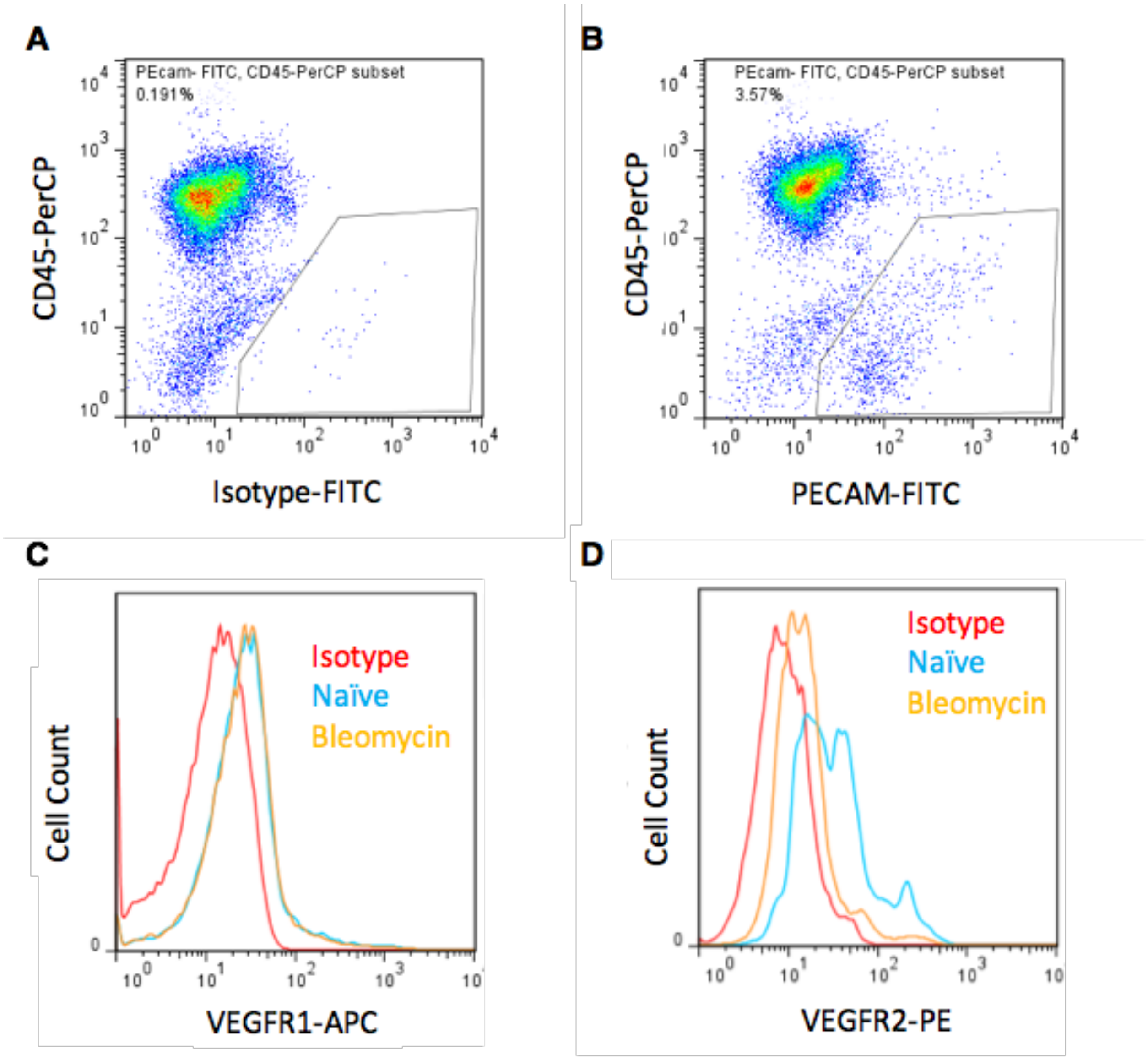

Supplemental figure 3. FACS gating strategy for measurement VEGF receptors on lung endothelial cells 48 hours after a single intratracheal dose of bleomycin. A. Staining with CD45-PerCP (Y axis) vs FITC isotype (X axis) is used to set the negative gate for the FITC channel. B. Staining with CD45-PerCP (Y axis) vs PECAM-FITC (X axis) identifies endothelial cells as CD45-PECAM+. C,D. Histogram of the selected endothelial cells stained for VEGFR1-APC (C) or VEGFR2-PE (D) determines the percentage of cells expressing either receptor in the naïve (blue) or bleomycin challenged lung (orange) based on the shift past the isotype control (red).

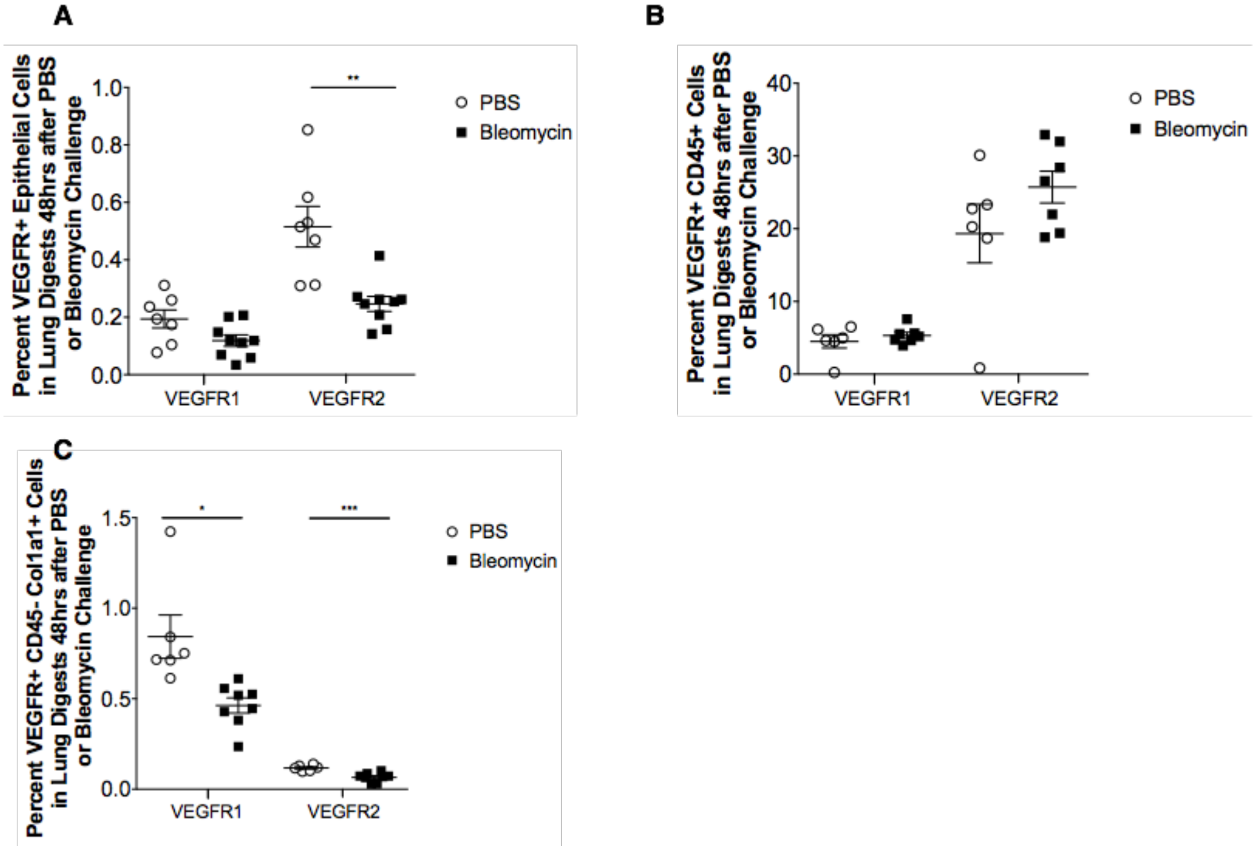

Supplemental figure 4. VEGF receptor expression on major lung cell populations 48 hours following intratracheal bleomycin exposure. Mice were given PBS control (open bars, n=5-7) or bleomycin (closed bars, n=7-9) and 2 days later killed and lung digests assessed by flow cytometry for VEGF receptor expression on epithelial cells (CD45- EpCAM+)(A), total CD45+ cells (B) and fibroblasts (CD45-, col1a1+; C). Bars represent mean ± s.e.m. *$p<0.05$, **$p\leq0.01$, ***$p\leq0.001$. Comparisons were made via Student's t test.

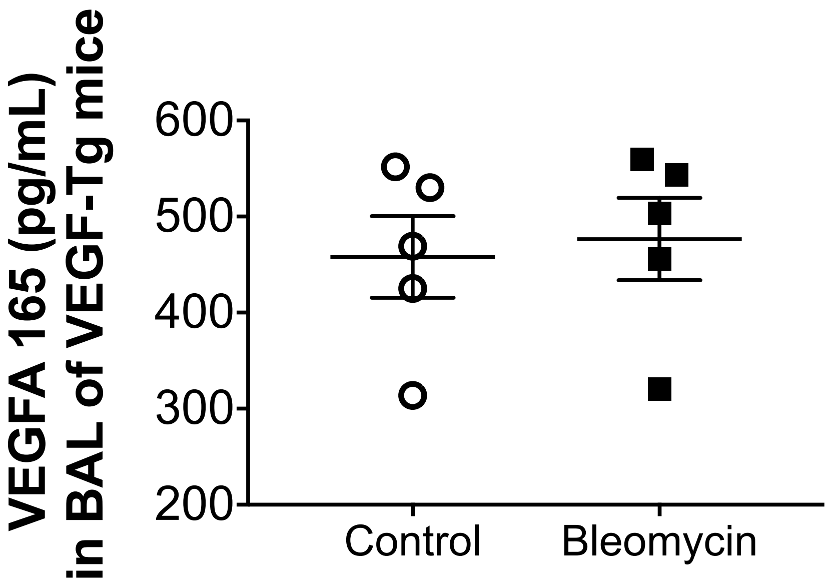

Supplemental figure 5. BAL human VEGF165 levels in wildtype and VEGF-Tg mice 14 days after intratracheal bleomycin challenge. BAL VEGF levels were measured by ELISA and bars represent mean ± s.e.m. No significant difference was observed when values were compared with Student's t-test.

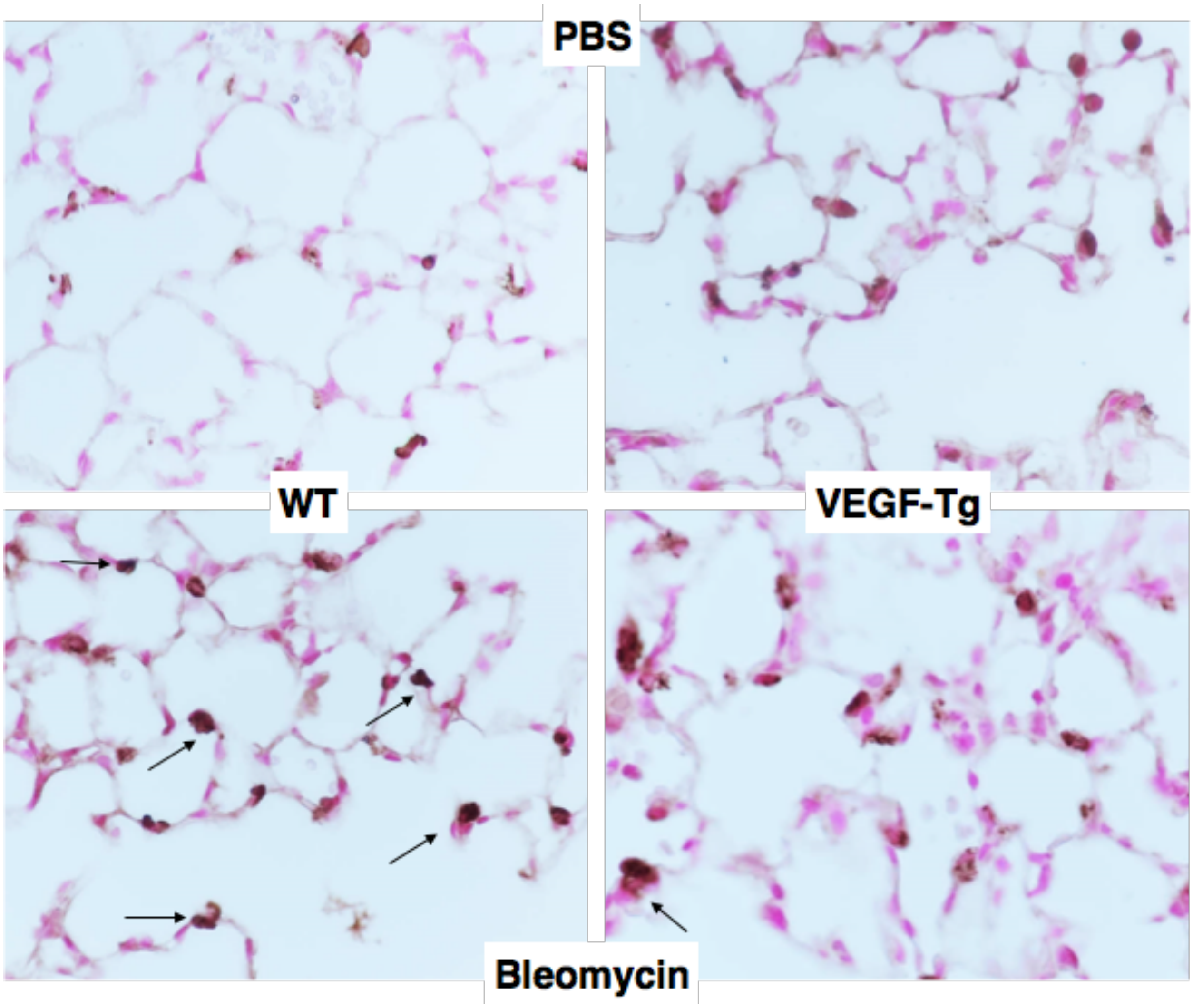

Supplemental figure 6. Combined detection of SPC (Brown) and TUNEL (black) in WT and VEGF Tg mice that did or did not receive bleomycin. The timepoint shown here is 48 hours. Arrows indicate cells that show co-detection of SPC and TUNEL. Images are 40X magnification and stained with nuclear fast red.

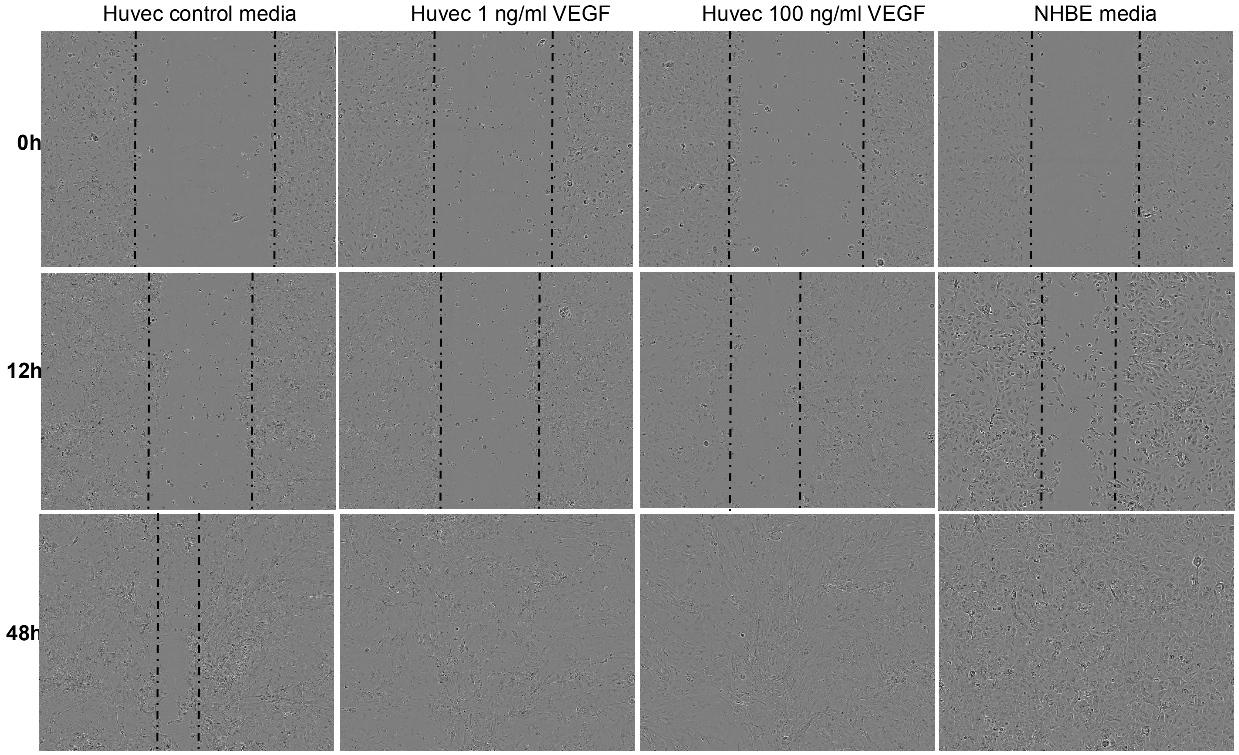

Supplemental figure 7. Pictorial representation of HUVEC conditioned media NHBE scratch assay. Dotted lines depict the region which was still uncovered by epithelial cells in the various conditions and the designated time points.

Supplemental Tables

Table S1. Epidemiological and clinical characteristics of the lung microarray cohort.

Characteristic	IPF (N=123)	Control (N=96)
**Age (yr) (Mean ± SD)**	64.8 ± 8.3	63.8 ± 11.2
**Gender, N (%)**		
**Males**	82 (66.7)	46 (47.9)
**Females**	41 (33.3)	50 (52.1)
**Race, N**		
**White**	113	88
**African-American**	5	3
**Hispanic**	1	2
**Asian or pacific**	2	1
**Native American**	0	1
**Not disclosed**	2	1
**Pulmonary function tests**		
**FVC%**	65 ± 16	95 ± 13
**DLCO%**	48 ± 18	83 ± 17
**FEV1%**	71 ± 16	95 ± 13

*FVC%: Forced vital capacity percent predicted, DLCO%: Carbon monoxide diffusing capacity percent predicted. FEV1% Forced expiratory volume in 1 second percent predicted.

Table S2. Characteristics of Progressive vs. Stable Subjects

	Progressive	Stable	P-Value
**N**	16	25	1.000
**Age (years)**			
**Mean ± SD**	70.99 ± 5.96	70.67 ± 7.23	0.883

Gender n (%)			
	Low VEGF<br>≤132.8 pg/ml	High VEGF<br>>132.8 pg/ml	P-Value
N	21	20	1.000
Race n (%)			
Age (years)			
Caucasian	15 (93.75)	25 (100)	1.000
Mean ± SD	69.37 ± 6.83	72.29 ± 6.35	0.16
Other	1 (6.25)	0 (0)	1.000
Gender n (%)			
Smoking Status n (%)			
Male	15 (71)	17 (85)	1.000
Ever/Current	11 (68.75)	21 (84)	1.000
Female	6 (29)	3 (15)	1.000
Never	5 (31.25)	4 (16)	1.000
Race n (%)			
FEV1	77.25 ± 18.79	81.88 ± 17.92	0.433
(% predicted mean ± SD)			
FVC<br>(% predicted mean ± SD)	70.13 ± 16.21	75.44 ± 12.41	0.243
DLCO<br>(% predicted mean ± SD)	37.63 ± 14.90	41.60 ± 10.68	0.326
GAP Index (mean ± SD)	4.75 ± 1.57	4.00 ± 1.16	0.086
VEGF pg/ml<br>(mean ± SD)	92.58 ± 58.32	197.2 ± 153.3	0.013

**Caucasian**	21 (100)	19 (95)	1.000
**Other**	0 (0)	1 (5)	1.000
**Smoking Status n (%)**			
**Ever/Current**	15 (71)	17 (85)	1.000
**Never**	6 (29)	3 (15)	1.000
**FEV1** **(% predicted mean ± SD)**	76.86 ± 17.66	83.45 ± 18.54	0.251
**FVC** **(% predicted mean ± SD)**	70.76 ± 16.25	76.10 ± 11.09	0.229
**DLCO** **(% predicted mean ± SD)**	40.81 ± 12.37	39.25 ± 12.86	0.694
**GAP Index (mean ± SD)**	4.24 ± 1.58	4.35 ± 1.14	0.797

Table S3. Characteristics of Subjects with Low vs. High VEGF
